# Effective peer-to-peer support for young people with end-stage renal disease: a mixed methods evaluation of Camp COOL

**DOI:** 10.1186/1471-2369-14-279

**Published:** 2013-12-21

**Authors:** Jane NT Sattoe, Susan Jedeloo, AnneLoes van Staa

**Affiliations:** 1 Centre of Expertise Innovations in Care, Rotterdam University, P.O. Box 25035, 3001 HA Rotterdam, the Netherlands; 2 Institute of Health Policy & Management, Erasmus University Rotterdam, P.O. Box 1738, 3000 DR Rotterdam, the Netherlands

## Abstract

**Background:**

The Camp COOL programme aims to help young Dutch people with end-stage renal disease (ESRD) develop self-management skills. Fellow patients already treated in adult care (hereafter referred to as ‘buddies’) organise the day-to-day program, run the camp, counsel the attendees, and also participate in the activities. The attendees are young people who still have to transfer to adult care. This study aimed to explore the effects of this specific form of peer-to-peer support on the self-management of young people (16–25 years) with ESRD who participated in Camp COOL (CC) (hereafter referred to as ‘participants’).

**Methods:**

A mixed methods research design was employed. Semi-structured interviews (n = 19) with initiators/staff, participants, and healthcare professionals were conducted. These were combined with retrospective and pre-post surveys among participants (n = 62), and observations during two camp weeks.

**Results:**

Self-reported effects of participants were: increased self-confidence, more disease-related knowledge, feeling capable of being more responsible and open towards others, and daring to stand up for yourself. According to participants, being a buddy or having one positively affected them. Self-efficacy of attendees and independence of buddies increased, while attendees’ sense of social inclusion decreased (measured as domains of health-related quality of life). The buddy role was a pro-active combination of being supervisor, advisor, and leader.

**Conclusions:**

Camp COOL allowed young people to support each other in adjusting to everyday life with ESRD. Participating in the camp positively influenced self-management in this group. Peer-to-peer support through buddies was much appreciated. Support from young adults was not only beneficial for adolescent attendees, but also for young adult buddies. Paediatric nephrologists are encouraged to refer patients to CC and to facilitate such initiatives. Together with nephrologists in adult care, they could take on a role in selecting buddies.

## Background

Young people with end-stage renal disease (ESRD) often achieve fewer developmental milestones and lag behind in development compared to both healthy peers and peers with other chronic conditions [1]. In general, the transition into adulthood is especially challenging for adolescents with chronic conditions, because they have to balance the usual developmental tasks with the medical challenges presented by the chronic condition [2]. Also, negative family exchanges like overprotection may hamper autonomy and self-advocacy development [3,4]. Young people with ESRD are known to be a vulnerable and unique group [5]. They are at risk for cognitive impairments, low educational attainment, and psychosocial and psychiatric problems [6-12]. Psychosocial development is closely linked to health-related quality of life and social participation [13]. Young adults who reached fewer developmental milestones in childhood and adolescence therefore experienced greater impact of their condition on their daily lives [13], while sound psychosocial development in early life was associated with successful social participation (e.g. [14]).

Since adolescence involves a shift from parental influences to peer relationships [15], and peers can provide psychosocial support [16,17] and influence treatment-related behaviours [18], creating opportunities for young people with chronic conditions to support each other is gaining popularity [19]. One popular method is the organisation of recreation camps. There is some evidence that participation in recreation camps has psychosocial benefits for children with chronic conditions. Various studies reported increased health-related quality of life [20-24], improved self-esteem, self-confidence, self-image or self-efficacy and sense of mastery [22,25-28], positive attitudes towards illness [29,30], increased disease-specific knowledge [26,31-33], and fostered independence, responsibility or self-management skills [26,33,34]. Yet, most studies have samples with an age range of 10–16 years on average [35], and further exploration of the benefits of participating in recreation camps for an older age group is needed [36,37]. Furthermore, relatively little is known about these camps’ working mechanisms [36,37], and there is a lack of qualitative or mixed-methods studies into participant experiences and the effects of recreational camping for young people with chronic conditions [35].

In the Netherlands, young people with ESRD can attend a yearly, nationwide one-week camp (Camp COOL) since 2007. Funded by the Dutch Kidney Foundation and private sponsors, the camp is free of charge for the young people. Paediatric healthcare professionals throughout the country refer patients to the camp. A unique feature is that fellow patients already treated in adult care (hereafter called ‘buddies’) organise the day-to-day program, run the camp and counsel the attendees, next to actively participating in the activities. Attendees are young people who still have to transfer to adult care. Only one other study reports on a more active role of adolescents with rheumatic disorders in organising and designing a camping program, but this more active role was not evaluated [28]. Our study aimed to explore the effects of this specific peer-to-peer support on self-management of all young people (16–25 years) with ESRD who participated in Camp COOL (CC) (hereafter called ‘participants’).

## Methods

### Study design & ethics

Epstein and colleagues [20] advocated the use of Mixed Methods Research (MMR) [38] to evaluate the effects of therapeutic camping for chronically ill, because the use of complementary quantitative and qualitative designs could lead to more enriched findings [20]. We used this method not only for this reason, but also because MMR was expected to contribute to the comprehensiveness and validity of the study [31,39]. The guidelines for Good Reporting of A Mixed Methods Study (GRAMMS) were followed [40], see Table 1. Quantitative measures such as questionnaires were combined with semi-structured interviews and participant observations during the camp weeks. Furthermore, different perspectives were explored by including healthcare professionals, buddies, attendees, and the initiators/staff of CC in the study sample. The qualitative component of our study adheres to the qualitative research review guidelines (RATS) [41].

**Table 1:** Guidelines for Reporting of A Mixed Methods Study (GRAMMS)*

	**Criteria description**
**1.**	Describe the design in terms of the purpose, priority and sequence of methods
**2.**	Describe the justification for using a mixed methods approach to the research question
**3.**	Describe each method in terms of sampling, data collection and analysis
**4.**	Describe where integration has occurred, how it has occurred and who has participated in it
**5.**	Describe any limitation of one method associated with the presence of the other method
**6.**	Describe any insights gained from mixing or integrating methods

*From: O’Cathain et al. 2008 [37].

More specifically, included in the study sample were: 1) all young people with ESRD that had once participated in CC during 2007–2010 (n = 52) or were visiting the camp in 2011 and/or 2012 (n = 38); 2) all paediatric nephrology professionals in the country that referred to CC (n = 5); and 3) the initiators/staff of CC (n = 4). The staff consisted of adults that stayed at the camp to assist the buddies in case they encountered problems they could not solve themselves. They kept themselves at the background and let the buddies run the camp.

The study was conducted in two consecutive phases, presented in Table 2. Participants were assured of confidentiality and data were processed anonymously. They received written information about the study and participants aged 12 years or older gave informed consent. Parents also provided informed consent for minors (<18 years). There were separate parts on the consent form for each of the study components (i.e. questionnaires, interviews and observations). The Medical Ethics Committee of the Erasmus MC University Medical Center approved all study procedures.

**Table 2:** Mixed methods research Camp COOL

***Study sample: Study phases:***	**Young people that participated in Camp COOL**	**Initiators or staff of Camp COOL**	**Nephrology professionals that refer patients to Camp COOL**
**Phase 1: Gaining insight into Camp COOL**	*February 2011*	*January 2011*	*January 2011*
	**Semi-structured interviews (n=2)**	**Semi-structured interviews (n=2)**	**Semi-structured interviews (n=3)**
	*March-June 2011*		
	**Retrospective questionnaire (n=24, response: 46%)**		
**Phase 2: Evaluation of Camp COOL in 2011 and 2012**	*September 2011, and October 2012*	*December 2012*	
	**Participant observations during camp**	**Semi-structured interviews (n=2)**	
	*September 2011, and October 2012*		
	**Pre-post questionnaires (n=36, response: 95%)**		
	*December 2011/2012, and January 2012/2013*		
	**Semi-structured interviews after camp (n=10)**		

### Phase 1: gaining insight into Camp COOL

The aims of phase 1 were:

1) To gain insight into the underlying principles of CC as an intervention for young people with ESRD, and the context in which it takes place. These insights were also used to develop our study materials for the evaluation of CC.

2) To pre-test our questionnaire and to gather preliminary information about the effects CC may have on participants.

### Semi-structured interviews

First, semi-structured interviews were held with the original initiators of CC (n = 2), with nephrology professionals referring patients to CC (n = 3), and with a buddy (n = 1) and an attendee (n = 1) who had participated in the previous camps (2007–2010). All original initiators and healthcare professionals were invited to participate, and were approached through e-mail. Initiators recruited former participants in this phase of the study.

For all interviews, topic guides were used. Professionals reflected on what they knew about CC, their rationale for referring patients to CC, the criteria used for selecting patients for CC, and their expectations considering the camp’s impact on both buddies and attendees. The initiators explained their aims for organizing CC, the concepts and ideas integrated in the program, and what they considered to be the camp’s impact on buddies and attendees. Former participants reflected on their experiences during CC and on the benefits.

### Questionnaire

Information from the semi-structured interviews with the initiators and healthcare professionals served as a basis for the retrospective questionnaire. A pilot version was tested in the interviews with the buddy and the attendee. Subsequently, all former participants (n = 52) were contacted by the initiators who sent out information letters and questionnaires by mail. Participants received three reminders: by mail (four weeks after initial invitation), by e-mail (two weeks after first reminder), and by phone (two weeks after the second reminder). Respondents were entered in a lottery to win one out of four vouchers worth €25. The questionnaire contained questions on participants’ background, self-management and participation and Camp COOL. The measured socio-demographic and disease-related characteristics [42], and the instruments used to measure general and disease-related self-efficacy [43,44], Health-related Quality of Life [45], and social participation [46], including their psychometrics are presented in Table 3. The questions specifically developed for this study and considering the influence of Camp COOL on the participants are presented in Additional file 1.

**Table 3:** Content and psychometrics of the measurement instruments (questionnaire)

	**Measured characteristics or constructs**	**Measurement instrument**	**Answer categories or scales**	**α**^**1**^
**Socio-demographics**	Age			
	Gender		Male/Female	
	Educational level		Low/High	
**Disease-related characteristics**	Age at diagnosis		0 years/1–5 years/6–12 years/13–16 years	
	Treatment type		Pre-dialysis/Haemodialysis/Peritoneal dialysis/Kidney transplantation/	
			Other	
	Limitations in mobility	Medical outcomes Study (MOS) 6-Items Short Form Health Survey [42]	3-point scale: 1 = severely limited/2 = somewhat limited/3 = not limited at all	.78
**Self-management and social participation**	General self-efficacy	10-item General Self-Efficacy Scale [43]	4-point Likert scale: 1 = not right/2 = hardly right/3 = somewhat right/4 = totally right	.71
	Disease-related self-efficacy	16-item^2^ On Your Own Feet Self-Efficacy Scale (OYOF-SES) [44]	4-point Likert scale:1 = yes certainly/2 = yes probably/3 = no probably not/4 = no, definitely not	.90
	Health-related quality of life	37-item European DISABKIDS condition generic questionnaire (DCGM-37) [45] with six domains: independence (I), social inclusion (SI), social exclusion (SE), emotion (E), physical (P), medication (M); and a general score (range: 0–100)	5-point Likert scale: 1 = often/2 = quite often/3 = sometimes/4 = almost never/5 = never	I: .86
				SI: .70
				SE: .85
				E: .81
				P: .46
				M: .79
	Social participation	Rotterdam Transition Profile (RTP) [46] with seven life areas: school/work, finances, (independent) living, (intimate) relationships, leisure, and mobility	Four transition (to adulthood) phases (0–3)^3^	na^4^
**Camp COOL**	Influence of living with the condition	10 items Effects of CC Scale See Additional file 1.	5-point Likert scale: 1 = completely disagree/2 = disagree/3 = somewhat agree/4 = agree/5 = completely agree	.92
	Value of peer-to-peer (i.e. buddy-to-attendee) support	Value of peer-to-peer support (2 items for buddies and 2 items for attendees) See Additional file 1.	5-point Likert scale: 1 = completely disagree/2 = disagree/3 = somewhat agree/4 = agree/5 = completely agree	
	Overall liking score for CC		10-point Visual Analogue Scale: 1 = lowest possible liking/10 = highest possible liking	

^1^α = Cronbach’s Alpha.

^2^This instrument originally consists of 17 items assigned to knowledge, coping and skills for hospital consultations. However, one item about expecting to be ready for the transfer to adult care was deleted, because it did not apply to our full sample.

^3^Young persons in phases 0 and 1 are still fully dependent on adults, e.g. parents, or display typical child behaviour. Young persons in phase 2 experiment with adult behaviour or orient to it. Phase 3 refers to full autonomy in participation. Because we were interested in successful transition to adulthood, the phases were dichotomised as follows: 0 = phases 0–2, 1 = phase 3.

^4^Construct validity was established in a previous study [45].

#### Phase 2: evaluation of Camp COOL in 2011 and 2012

The aims of phase 2 were:

1) To gain insight into the effect of peer-to-peer support as working mechanism of CC.

2) To study the effects participating in CC has on self-management of young people with ESRD.

Prior to the camp, participants of the camp in 2011 and 2012 received a letter informing them about the research and asking for their consent, and in case of minors for parental consent as well. They filled out an informed consent form, agreeing to all research methods.

### Observations

Participant observations were conducted to gain insight into the establishment of peer-to-peer support during CC. Participants received information before the camp and provided consent. Two researchers (JS & SJ) and four trained nursing and physical therapy students observed participants during CC 2011 and CC 2012 and were introduced during the first activity of CC. They took field notes and filled out structured forms about participants’ attitudes and behaviour, and topics discussed. Special attention was paid to buddy-attendee interaction. Other broad themes on the forms were: general description of the event (e.g. description of the setting and format), topics addressed during the event, interaction between participants, and other notable happenings. Observers wrote down their findings per theme in narratives. Some activities required the group to be split into smaller groups. Therefore, to be able to observe the same activity in different groups, three to four observers were present at CC 2011 and CC 2012. At least one of the researchers teamed up with the trained students during observations, and the observers were present at every activity or event.

### Semi-structured interviews

Two staff persons were interviewed at the campsite in 2011 and 2012. They talked about the daily programme of CC and about the perceived impact of CC on buddies and attendees. They were selected because they were the only staff persons not interviewed during phase 1. All participants had been requested to indicate their willingness to participate in semi-structured interviews performed 4–12 weeks after the camp. Ten participants who attended CC 2011 or CC 2012 (31.3%) were willing to participate and were subsequently interviewed. They reflected on their experiences, the different elements of the program, the buddy-to-attendee support, and the benefits of participating in CC.

### Pre-post questionnaires

All participants of the camps organised in 2011 or 2012 (n = 38), filled out pre-post questionnaires containing questions similar to the ones in the retrospective questionnaire. In the pre-questionnaire, administered at camp start (T0), the questions considering the camp experiences had been rephrased to reflect expectations. The post-questionnaire, administered at camp closure (T1), asked after outcomes of these expectations.

#### Data analyses

Interviews were all digitally recorded and transcribed ad verbatim. The interview transcripts and the observation forms were imported into separate files in the qualitative software package Atlas.ti 6.2. (http://www.atlasti.com). Thematic analysis was applied on both data sets, and data from different parties (buddies versus attendees, and participants versus initiators/staff) were constantly compared. In Atlas.ti, initial codes (themes) were formulated on the basis of the interview guides and the observation form. These were complemented with newly formed codes. Broad themes were derived from the interview guide, while subthemes were empirically derived from the data. Themes for instance considered ‘going to CC, ‘at the camp’ , ‘peer-support’ and ‘CC and transition to adulthood/adult care’. Subthemes were for example ‘reasons to participate’ , ‘value of participating’ , ‘programme elements’ , ‘buddies’ , and ‘becoming independent’.

SPSS 20.0 (SPSS Inc., Chicago, IL) was used for all the statistical analyses. Means, standard deviations and proportions were used for descriptive analyses. Non-parametric tests were used for pre-post analyses. Finally, effect sizes were estimated for significant differences (Cohen’s d).

#### Validation & integration

Method triangulation and peer-review enhanced validation for the qualitative findings. Two researchers (JS & SJ) discussed all preliminary analyses of the observations and interviews; the final analyses were presented to and discussed with the supervisor (AvS) and the members of the advisory board. Validation for the quantitative findings was enhanced through pre-testing the questionnaire with one buddy and one attendee. None of the respondents had difficulties in answering the questions, but they had some useful suggestions considering the formulation of questions. Filling out the questionnaire took approximately 20 minutes.

Findings from the MMR were integrated in different ways. First, the qualitative findings from Phase 1 were summarised and used to develop the questionnaires. Also, statistical comparison of first phase quantitative results with the second phase quantitative results led to integration. Final integration was achieved through comparing the qualitative and quantitative findings of both phases, and drafting this manuscript.

## Results

First, we present the final study samples. Then, the origins and goals of CC are presented to enhance understanding of CC as intervention for young people with ESRD. This section is based on the results from the interviews with initiators and healthcare professionals. Next, the results from the observations, interviews with all three parties, and questionnaires are presented. The findings are integrated in the last paragraph.

### Study samples

In the two phases, 19 respondents were interviewed:4 initiators/staff, 3 healthcareprofessionals, 6 buddies, and 6 attendees (Table 4). Buddies were on average 21 years old (range: 18–25 years), while for attendees this was 17 years (range: 16–18 years).

**Table 4:** Characteristics of interviewed respondents

**Respondent code**	**Type of respondent**	**Gender**	**Attendance at camp COOL**
A	Initiator (Parent)	Female	Yes
B	Initiator (Paediatric nephrologist)	Male	Yes
C	Paediatric nephrologist	Female	No
D	Social worker	Female	No
E	Social worker	Male	Yes
F	Buddy	Female	4 x buddy
G	Attendee	Female	2 x attendant
H	Buddy in 2011	Female	1 x buddy, 1 x attendant
I	Buddy in 2011	Female	2 x attendant
J	Buddy in 2011	Male	2 x buddy
K	Attendee in 2011	Male	First time
L	Attendee in 2011	Male	First time
M	Attendee in 2011	Female	First time
N	Buddy in 2012	Female	3 x buddy, 2 x attendant
O	Buddy in 2012	Female	1 x attendant
P	Attendee in 2012	Female	First time
Q	Attendee in 2012	Male	First time
R	Staff	Male	Yes
S	Staff	Female	Yes

In Phase 1, 24 out of 52 former participants (46%) filled out the retrospective questionnaire. Most of them were girls, and had received kidney transplantation. Mean age of the respondents was 20.8 (±3.2) years, and half of them had been attendees only; while the other half had been both attendees and buddies. Background and self-management characteristics are summarised in Table 5.

**Table 5:** Characteristics of questionnaire respondents: n (%) or mean (±SD)

	R* (n = 24)	T0* (n = 32)	T1* (n = 32)	***p; *** Cohen’s d**
**Background**				
**Age** (at time of questionnaire) [15-29]^	20.8 (±3.2)	19.1 (±2.4)		
**Gender** (male)	8 (33.3)	17 (53.1)		
**Educational level** (high)	8 (50.0)^1^	11 (39.3)^4^		
**Age at diagnosis**				
*0 years*	11 (45.8)	15 (46.9)		
*1*–*5 years*	3 (12.5)	8 (25.0)		
*6*–*12 years*	5 (20.8)	5 (15.6)		
*13*–*16 years*	5 (20.8)	4 (12.5)		
**Treatment type**				
*Pre-dialysis*	-	2 (6.3)		
*Haemodialysis*	4 (16.7)	6 (18.8)		
*Peritoneal dialysis*	-	-		
*Kidney transplant*	20 (82.3)	18 (56.3)		
*Other*	-	6 (18.8)		
**Limitations in mobility**[6-18]^	7.9 (±2.0)^2^	7.6 (±2.0)^5^		
**Self-management**				
**General self-efficacy**[10-40]^	27.7 (±3.0)	30.7 (±4.5)	32.1 (±4.7)	<.05; .31
**Disease-related self-efficacy**				
Coping domain [4-16]^	14.3 (±1.9)	13.8 (±2.3)^7^	13.7 (±2.0)^2^	ns
Knowledge domain [6-24]^	22.0 (±2.1)	21.7 (±2.6)^9^		
Skills for hospital consultations [6-24]^	21.3 (±3.5)	20.8 (±3.2)^11^	21.5 (±2.5)^10^	ns
**HRQoL [0–100]^**				
General HRQoL	73.9 (±11.4)	72.4 (±17.0)^2^	72.1 (±14.2)	ns
*Independence domain*	82.9 (±14.0)	78.1 (±13.2)	83.9 (±15.0)	<.01; .44
*Emotion domain*	63.2 (±13.5)	71.1 (±23.3)^5^	71.3 (±18.4)^2^	ns
*Social inclusion domain*	75.7 (±14.1)	74.1 (±18.9)^6^	70.5 (±15.8)	<.05; -.19
*Social exclusion domain*	77.4 (±18.8)	77.1 (±17.6)^5^	75.2 (±18.2)^2^	ns
*Physical domain*	68.2 (±15.9)	60.6 (±19.4)	60.1 (±16.4)^2^	ns
*Medication domain*	77.9 (±16.4)	71.0 (±20.4)^2^	72.2 (±21.7)^2^	ns
**Autonomy in social participation** (yes independent)				
*Finances*	14 (58.3)	3 (15.0)^5^		
*Employment*	7 (29.2)	3 (15.0)^6^		
*Living*	6 (25.0)	3 (15.0)^6^		
*Relationships*	15 (65.2)^2^	16 (80.0)^6^		
*Sexuality*	11 (50.0)^3^	9 (52.9)^10^		
*Transportation*	22 (100)^3^	14 (70.0)^6^		
*Leisure*	17 (70.8)	13 (68.4)^5^		

*R = retrospective; T0 = pre-camp; T1 = post-camp.

^Theoretical range.

**Wilcoxon Signed Ranks Test (paired) for differences between T0 en T1 measurements, and Cohen’s d for effect sizes.

Missing values: ^1^n = 8, ^2^n = 1, ^3^n = 2, ^4^n = 4, ^5^n = 13, ^6^n = 12, ^7^n = 3, ^8^n = 1, ^9^n = 22, ^10^n = 21, ^11^n = 9.

In Phase 2, 38 participants of CC in 2011 and/or 2012 were asked to fill out pre and post questionnaires. Four attended both camps and filled out the questionnaires twice. Only the data from 2011 were used for the analysis, because this was their first experience with CC. Two respondents did not fill out the post questionnaire, because they had left to undergo treatment. Consequently, the pre-post sample consisted of 32 (84%) young persons with ESRD. Most of them were boys, and had kidney transplantation. Mean age was 19.1 (±2.4) years. Background and self-management characteristics are summarised in Table 5.

Participants were observed during CC 2011 and 2012; in total on 8 out of 10 days. The programme elements observed are presented in Table 6.

**Table 6:** Program elements Camp COOL 2011 and 2012

**CC-2011**	**CC-2012**
Workshop ‘Present yourself’	Theater performance by professional artists on transition to adulthood (in general)
Workshop ‘Present yourself’	Art workshop, creating a self-portrait
Movie making workshop & self-made movie about Rating Camp COOL	Drumming workshop
Dancing (Zumba) workshop)	Acting workshop & self-made talk show about transition, independence, and living on your own
Sports	Free time
Cook/ing teams	
Free time	

### Camp COOL: the intervention

#### The rationale behind Camp COOL

One of the initiators had heard about a ‘transition camp’ in the UK [28] and felt this approach might be helpful for young people with ESRD in the Netherlands as well. He discussed his idea with parents and fellow professionals, and together they explored the specific needs of young people with ESRD. Realising that acquiring autonomy and independence was especially hard for these young people, they widened the scope of the camp (particularly preparing for transition from paediatric to adult care and self-care) to a self-management camp (aimed at independent living with ESRD, i.e. the transition to adult care and adulthood). *“Self-management is the main theme of Camp COOL. It […] requires self-confidence, self-efficacy, and self-consciousness”* (A). Next to this, knowledge of the disease and various skills are important for self-management.

Acquiring self-management skills was facilitated by buddy-to-attendee support. This implied that buddies –fellow patients already gone through the transition to adulthood and adult care – lead the day-to-day program, run the camp and counsel the attendees who have not moved on to adult care yet. Initiator A explained: *“They manage the week. We are present, but are invisible. We are only available if there is really something they need to know. But even then, we always let them come up with their own solutions first and ask them what they think is needed to solve a problem.”* The concept of buddy-to-attendee support presupposes that buddies will share their lived experiences, allowing for transfer of experiential knowledge. Also, it is hoped that buddies become role models. Buddies are not formally selected or trained, but receive some coaching during the two days before start of the actual camp. Also, buddies have a ‘buddy meeting’ every day to discuss anything that requires attention. Initiators select former attendees and ask them to become buddies, but apply no explicit selection criteria.

Furthermore, the programme elements support building general competencies, e.g. a ‘how to present yourself’ workshop. There are no activities focussed on the disease; attendees will not be lectured about side effects, for example. Although buddies lead the day-to-day program, in 2011 the initiators/staff had pre-selected the programme elements. However, in 2012, the buddies had more to say about the programme by selecting specific elements, presented in Table 5. This was done as a first step to evolve the buddy role, because it was noticed in the past years that buddies benefited from this role. In both years, a hospital social worker and an initiator were present.

#### The referring role of healthcare professionals

C (nephrologist) defined her referring role as being a *“counsellor”* who *“recruits young people”* with ESRD. Furthermore she mentioned that professionals may be asked to take over the *“background”* role of the initiators during the camp, *“only interfering when needed”*.

All professionals agreed that age was the major selection criterion; 16 years or older in general. A social worker added that she also considers impact of the condition on the person’s daily life: *“Especially those who daily take medication and are on a diet. Or those who do not know how to deal with the condition at school, and those who have yet to learn to become independent”* (D).

### Observations during the camp

Notably the first-timers needed to get acquainted with the new people they met and with the camp’s routine. Buddies helped breaking the ice. They started conversations with attendees, encouraged attendees to talk with one another, and told a lot about themselves to create an open atmosphere. There was an observable difference between first-timers and attendees who had joined previous camps. The latter were less hesitant to interact with others, and less often relied on their buddies. Buddies proactively engaged the new attendees in conversations. As the first day progressed, the ice had melted, and there was a warm and relaxed atmosphere.

Participants talked a lot with each other during activities and free time, a great deal about medical and social aspects of ESRD. Side effects of medication were discussed, in particular Prednisone. Insomnia, feeling hungry, and a *“fat head”* were often mentioned as annoying side effects. Participants during CC 2011 even came up with a story about a *“Prednisone park”* when they presented a show as one of the activities. Still, participants joked a lot about side effects. Other medical topics were transplantation, diets, treatment frequency, and treatment options. Social topics addressed were school, work, sports, risky behaviours like smoking, drinking or doing drugs, but also dealing with ESRD in social life. A major issue was the influence of ESRD and its treatment on school carrier, i.e. either or not being enrolled in special education and whether they felt pushed by their environment to do so. Another hot topic was ‘how to become independent from parents’. Participants during CC 2012 presented this in their evening show.

During certain activities the buddy role was more prominent, for instance during the ‘Present yourself’ workshop and the acting workshop. Buddies encouraged the attendees to actively participate in workshops. During the moviemaking workshop, one of the buddies urged attendees to come up with ideas: *“Hello, listen, I’m talking all the time here. You guys could come up with something as well!”* During free time, the buddy role varied from telling their attendees it was their turn to do the dishes to reminding them of their diets.

The buddy role was less prominent in the art workshop and preparations for the evening show. Here, the buddies seemed to adopt a more passive role and let the attendees figure things out on their own. In the preparations for the evening show, they only offered ideas on how the selected themes should be presented. Consequently, the show was largely the work of the attendees.

### Interviews: the value of Camp COOL

All interviewed parties acknowledged that young people with ESRD needed to be supported in their development of self-management. Professionals mostly emphasised that young people with ESRD in adult care tended to show lack of independence, and initiators held the opinion they should actively develop autonomy and readiness for adult care and adult life. A former buddy (F) reasoned that adult care requires certain skills that are not necessarily trained for in paediatric care: *“You have to be attentive yourself. In paediatric care they arranged everything for you […]. You must ensure that they won’t just let you be. This happens. Other buddies had the same experience.”*

Buddies and attendees had different reasons to participate in CC. While buddies thought of CC as a place to meet the others again and to enjoy themselves, attendees in general had to be encouraged by their parents to join. *“At first, I wasn’t really up for it. My father signed me up. But I did not regret going to Camp COOL”* (M).

The most valued aspect of CC was peer support. Participants did not only appreciate the informative or instructional character of the peer support, but also found that meeting others *“who have been through the same”* helped them to *“put”* themselves and their ESRD *“into perspective”*. J (buddy) explained: “*Realising that you are not the only one, or even that your own condition is not as bad as that of others. For instance, I saw that I was not the only one that got tired easily during sports.”* Social comparison seems to be an inherent part of peer activities, as mentioned by K (attendee): *“Well, having heard stories of others, I feel lucky that things aren’t going that bad for me. Some said they have been on dialysis for years or are still waiting for kidney transplantation. Yes, I think I am lucky that I do not have to wait anymore.”* Young people emphasised that contacts with others in their social network differed from contacts with peers with ESRD: *“Other ESRD patients will understand your condition better than your own family or friends”* (L). N gave specific examples: *“The freedom to take your medication without anyone asking you why you have to do this. And, that you do not have to hide a shunt from the outside world.”*

Participants particularly appreciated the informative character of peer support. The sharing of experiences gave them new information on dealing with healthcare professionals, treatment options, and possible side effects. M (attendee) said: *“I didn’t even know that I had side effects. […] I sat down and said I was hungry again. And they said ‘Prednisone’. I asked: ‘Prednisone?!’ And they said, yes, [being hungry] is one of the side effects of Prednisone. I went like, side effects?!”* Young people also learned more about generic issues of living with ESRD. P (buddy) mentioned living independently as an example: *“I learned something about being independent, because we talked about living on your own and how to arrange for that to happen.”* Other issues mentioned were school, work, and dealing with friends.

Finally, buddies and attendees ascertained that the programme elements had helped them to develop more *“self-confidence”* and *“perseverance”*, and had made it easier for them to *“be more daring”* and *“open towards others”*. The healthcare professionals, however, were less certain about the exact effects of CC. *“I cannot imagine it having no effects at all. Still, I can’t specifically point out what the effects are”* (E). Their reluctance was related to the question whether or not any positive effects were directly attributable to the camp.

### Interviews: buddy-to-attendee support

The buddy is an important part of CC, and was much appreciated. The attendees mostly viewed the buddy as a companion who helped them through the first day and who guided the activities. *“I think it is important to have a buddy when you first get there. That he or she helps you to get used to the new environment. I had a very experienced buddy, who told me a lot”* (L, attendee). They appreciated that they could learn from their buddies, because: *“A buddy is more experienced [in living with ESRD]. So, it’s a good thing that he is here. […] A doctor can tell you all of it, but doesn’t experience things. A buddy does”* (Q, attendee).

The initiators noticed that buddy-to-attendee support did not only benefit attendees, but that buddies themselves grew wiser from managing the camp too. *“The responsibility for the camp and the attendees makes them grow”* (B). Buddies in general indeed described having *“responsibility”* as the most important aspect of their role as and found this role to be threefold: 1) looking after others, 2) giving advice to others, and 3) running the program. The supervising role relates to monitoring medical regimen adherence, but also seeing to it that the attendee feels well and enjoys the activities. “*Especially the medication, she tried to hold off taking them. So, I tried to convince her it’s crucial to take it on time”* (N, buddy). Buddy O said this about her attendee: *“You almost had to feed her. I really had to take care she ate enough; I sort of had to force her to do so.”*

The advisor role revolves around listening to the attendees’ stories and being able to advise them if asked to. Questions often concerned living with ESRD but could be medically oriented as well. Buddy O, for example, was asked about types of dialysis: *“I did both types of dialysis and therefore could tell them about the differences and consequences of choosing one method over the other”* (O). Finally, smooth running of the programme is the responsibility of the buddies in their leader role: *“We as buddies take care of the daily camping program, we lead the camp”* (J).

All buddies mentioned that being a buddy was fruitful for them: they learned a lot and it increased their self-confidence. However, some felt insecure at times. Buddy N said: *“I found that difficult, because I could understand her feelings [of being misunderstood by family and friends], and of course I can advise her, but it made me feel like a psychologist and that is not my task”*. This goes to show that the buddy role is a challenging one. Buddy O had come to realise this: *“I do not get angry easily, but sometimes that’s what is needed. So, if someone is extremely annoying, I would not know how to deal with it”*. Fortunately, the buddies would work together if needed and discuss problems during the buddy meeting.

### Quantitative results: self-management of young people with ESRD and pre-post effects of Camp COOL

On average, all participants scored relatively high on self-efficacy measures and on health-related quality of life (Table 5). As for social participation, most of the respondents still lived with their parents (respectively 75% and 85% in the retrospective and 2011–2012 groups), and were involved in a romantic relationship (65.2% and 80.0%). Also, half of them or more were independent in the areas of sexuality (50.0% and 52.9%), transportation (100% and 70%), leisure (70.8%), and 68.4%). The young adults in the retrospective group were more frequently financially self-supporting (58.3%) than the participants in 2011–2012 (15.0%) (Table 5).

The 2011–2012 group reported significantly higher general self-efficacy after CC (Cohen’s d = .31; p < .05). Disease-related self-efficacy did not differ between the T1 and T0 assessments. The mean score on the independence domain after CC was significantly higher (d = .44; p < .01), but the mean score on the social inclusion domain was significantly lower (d = −.19; p < .05) (Table 5). Discriminating between buddies and attendees, only attendees reported a significantly higher score on general self-efficacy (d = .37; p < .05) after CC. Also, only attendees perceived significantly lower HRQoL on the social inclusion domain after CC (d = −.33; p < .05). Buddies reported significantly higher HRQoL on the independence domain afterwards (d = 1.1; p < .05) (Table 7).

**Table 7:** Buddy-attendee comparison: n (%) or mean (±SD)

	**Buddies (n = 18)**		p; Cohen’s d**	**Attendees (n = 14)**		p; Cohen’s d**
	T0*	T1*		T0*	T1*	
**Background**						
**Age**	20.7 (±2.0)			17.1 (±1.1)		
**Gender** (male)	10 (55.6)			7 (50.0)		
**Educational level** (high)	7 (50.0)^5^			4 (28.6)		
**Age at diagnosis**						
*0 years*	9 (50.0)			6 (42.9)		
*1*–*5 years*	5 (27.8)			3 (21.4)		
*6*–*12 years*	1 (5.6)			4 (28.6)		
*13*–*16 years*	3 (16.7)			1 (7.1)		
**Treatment type**						
*Pre-dialysis*	1 (5.6)			1 (7.1)		
*Haemodialysis*	5 (27.8)			1 (7.1)		
*Kidney transplant*	11 (61.1)			7 (50.0)		
*Other*	1 (5.6)			5 (35.7)		
**Limitations in mobility**[6-18]^	7.0 (±2.0)^1^			7.8 (±2.0)^2^		
**Self-management**						
**General self-efficacy**[10-40]^	31.2 (±4.1)^2^	32.1 (±4.2)^2^	ns	30.2 (±5.1)^2^	32.1 (±5.6)^3^	<.05; .37
**Disease-related self-efficacy**						
*Coping domain [*[4]*-*[20]*]^*	14.4 (±1.8)^3^	13.8 (±1.8)	ns	13.1 (±2.8)^2^	13.6 (±2.4)^2^	ns
*Knowledge domain [*[7]*-*[35]*]^*	26.2 (±2.9)^4^	25.8 (±3.1)^1^	ns	23.6 (±3.3)^4^	24.2 (±2.8)^4^	ns
**HRQoL** [0–100]^						
*General HRQoL*	73.3 (±13.2)^1^	74.0 (±11.6)^2^	ns	72.0 (±18.9)^2^	69.8 (±16.9)	ns
*Independence domain*	77.9 (±7.4)	86.1 (±10.9)	<.05; 1.1	78.5 (±18.9)^2^	81.0 (±19.0)	ns
*Emotion domain*	66.7 (±20.7)^1^	73.1 (±15.1)^2^	ns	73.2 (±24.9)^2^	69.1 (±22.2)	ns
*Social inclusion domain*	72.2 (±12.0)^1^	72.5 (±11.8)	ns	74.9 (±21.5)	67.9 (±20.0)	<.05;-.33
*Social exclusion domain*	83.8 (±15.8)^1^	79.4 (±15.7)^2^	ns	74.0 (±18.2)^2^	70.1 (±20.4)	ns
*Physical domain*	59.9 (±13.7)	58.7 (±11.7)	ns	61.6 (±25.5)	62.2 (±21.8)^2^	ns
*Medication domain*	75.3 (±17.5)	75.0 (±20.3)^2^	ns	65.2 (±23.3)^2^	68.8 (±23.6)	ns
**Overall score for CC**[1-10]^		9.2 (±.73)			8.4 (±.68)	

*T0 = pre-camp; T1 = post-camp.

**Wilcoxon Signed Ranks Test (paired) for differences between T0 en T1 measurements, and Cohen’s d for effect sizes.

Missing values: ^1^n = 12, ^2^n = 1, ^3^n = 2, ^4^n = 13, ^5^n = 4.

^Theoretical range.

A reasonably large proportion of respondents, i.e. half or more, found that participating in CC had positively influenced their daily lives on several areas, e.g. attitude toward illness, independence, self-confidence, ability to socially interact with others, knowledge of the condition, and insight into what the transition to adult care involves. The least influence was perceived on healthier living (respectively 16.7% and 37.5% in the retrospective and 2011–2012 groups) (Table 8). The majority of the attendees appreciated having a buddy (91% and 85.7%), but the ‘personal’ buddy was not always the one they learned the most from. More than half of the buddies in the 2011–2012 group (57.2%) thought they learned more from being a buddy than from being an attendee, but in the retrospective group fewer buddies agreed with this statement (28.6%). The majority in both groups would recommend being a buddy to others. The mean (±SD) overall CC appreciation score assigned by participants in the retrospective group was 8.0 (±1.2) on a scale from 1 to 10, versus 8.9 (±.82) by participants in the 2011–2012 group. Respondents in the 2011–2012 group were also more positive about the perceived effects of CC on dealing with physical limitations, attitude toward illness, and knowledge of the condition than those in the retrospective group (Table 8). There were no significant differences between expectations and outcomes in the 2011–2012 group.

**Table 8:** Rating Camp COOL: frequency (%) of respondents agreeing or totally agreeing with the statements; mean(±SD) for overall score

	R* (n = 24) outcomes	T0* (n = 32) expectations	T1* (n = 32) outcomes
**I expect (T0) / found (R and T1) CC to positively influence my:**			
*Dealing with physical limitations*	9 (37.5)**	21 (65.6)	21 (65.6)**
*Attitude toward illness*	11 (45.8)***	19 (59.4)	24 (75.0)***
*Healthier living*	4 (16.7)	8 (25.0)	12 (37.5)
*Knowledge of the condition*	9 (37.5)**	20 (62.5)	18 (56.3)**
*Independence*	7 (29.2)	21 (65.6)	16 (50.0)
*Self-confidence*	11 (45.8)	16 (50.0)	16 (50.0)
*Ability to socially interact*	10 (41.7)	12 (37.6)	16 (50.0)
*Insight into what the transition to adult care holds*	10 (43.5)^1^	19 (61.3)^1^	18 (51.3)
*Being prepared for transition to adult care*	7 (30.4)^1^	15 (62.5)^5^	12 (52.2)^1^
*Assertiveness*	8 (33.3)^1^	11 (35.5)^1^	14 (43.8)
**The value of buddy-to-attendee support (yes):**			
*As an attendant, I appreciated having a buddy*	10 (91.0)^2^		12 (85.7)^6^
*As an attendant, I learned the most from my buddy*	5 (45.5)^2^		8 (57.2)^6^
*As a buddy, I learned more during CC than I did as attendant*	2 (28.6)^3^		8 (57.1)^4^
*As a buddy, I would recommend being a buddy to others*	8 (80.0)^4^		15 (93.8)^7^
**Overall score for CC**[1-10]^	8.0 (±1.2)		8.9 (±.82)^1^

*R = retrospective; T0 = pre camp; T1 = post camp.

^Theoretical range.

**p < .05; Wilcoxon Signed Ranks Test (independent) for differences between R and T1 (at mean level).

***p < .01; Wilcoxon Signed Ranks Test (independent) for differences between R and T1 (at mean level).

Missing values: ^1^n = 1, ^2^n = 13 (attendees only), ^3^n = 17 (buddies only), ^4^n = 14 (buddies only), ^5^n = 8, ^6^n = 18 (attendees only), ^7^n = 16 (buddies only).

### Integration of findings

The 2007–2010 and 2011–2012 groups were very similar when considering HRQoL and social participation. The first group was more financially self-supporting, but then, their mean age was higher at time of the questionnaire. All parties acknowledged that young people need support in their development of self-management. This was also implicitly observed during the camp: becoming independent was a hot topic, and was processed in activities by the campers.

The perceived effects of CC mentioned in the interviews were increased self-confidence, more knowledge of ESRD, feeling capable of being more responsible and open towards others, and daring to stand up for yourself. In the quantitative evaluation of CC half or more of the participants reported the same effects. Furthermore, the pre-post analyses showed that general self-efficacy of attendees, and independence as domain of HRQoL of buddies had increased after attending CC, whereas social inclusion as domain of HRQoL of attendees had decreased. Peer support was the most valued aspect of CC, both mentioned in the interviews and found in the questionnaires. It was perceived as informative, but even more importantly as a great opportunity to meet fellow patients. This was also observed during CC.

Appreciation of buddy-to-attendee support was demonstrated in both the interviews and questionnaires. Buddies were expected to transfer knowledge and to be an example for attendees. Indeed, during the interviews attendees mentioned that they learned a lot from buddies, and observations showed the same. Buddies shared experiences and knowledge, looked after their attendees, and led the camp. The buddy role was given shape as a pro-active combination of supervisor, advisor, and leader.

## Discussion

### Self-management support, effects of CC, and the buddy role

It would seem evident that young people with ESRD need support in developing self-management skills. When it comes to social participation, for instance, young people in our samples most resemble those we labelled as “outgoing laggers” in another study, with little autonomy in the areas of finances, employment, and living, while at the same time enjoying romantic relationships and socialisation with peers [47]. Becoming independent in the areas of living, employment and finances was much discussed during CC, showing that young people with ESRD seem to be lagging behind in these areas. This finding is in line with the results of other studies [6,7], and calls for more specific support for work-participation. The different attitude towards self-management found for the majority of the older participants, despite similar HRQoL and social participation, indicates that age is an important determinant of self-management.

The positive effects we encountered – e.g. increased self-efficacy, self-confidence, and knowledge of ESRD – were also reported previously as benefits of therapeutic camping for young people with a variety of chronic conditions [20,25-27,31,33,48,49], and benefits of peer support [16]. It seems that Camp COOL creates an environment that allows for “mastery experiences” and “learning by examples” [50]. Greater self-efficacy can positively affect different levels of functioning in young people with ESRD. This is especially valuable for those who still have to transfer to adult care and adulthood, and provides support to paediatric nephrologists for referring young people to CC or initiating such camps.

However, we also found diminished sense of social inclusion (as part of HRQoL) of attendees after CC. This may be due to the fact that a subculture is created during the camp in which the attendees perceive themselves as being different from others. This was identified in previous studies as a possible disadvantage of peer support [19], and requires attention. Olsson and colleagues [19] argued that this “over-identification” might be counteracted by addressing it in the group. This may be an important recommendation for future camps.

Participating as a buddy during CC had a positive effect on the independence domain of HRQoL, implying that being a buddy fosters confidence in future living without impairments caused by ERSD. Positive effects of a challenging buddy role have been reported previously for renal peer support volunteers [51], and peer leaders in an asthma self-management camp [52]. Also, the buddies’ combined roles of supervisor, advisor and leader for seems to match with the three types of assistance identified with peer support based on experiential knowledge (i.e. emotional, appraisal and informational assistance) [53,54]. Still, this combined role might be too challenging for untrained buddies. Although buddies receive some coaching and have buddy meetings, for the buddy role to be effective a buddy should possess the skills and knowledge required to act as a role model [55]. Selection and training of peer supporters is important. Therefore, a recommendation for CC in the future is to more carefully select buddies and to specifically train or coach them to be models. This could counteract any negative effects of peer support [16,19]. Paediatric nephrologists could involve their counterparts from adult care in selecting potential buddies.

### Strengths and limitations

This study is one among the first to evaluate therapeutic camping for young people with ESRD and one of the few considering effects of therapeutic camping in chronically ill young people in MMR. To our knowledge, it is the first that more specifically looks at the benefits of buddy-to-attendee support during therapeutic camping. Furthermore, the use of MMR added to the comprehensiveness of this study, and led to a broader insight into CC. Mixed methods research also partially overcomes the disadvantage of a convenience sample and of the small sample size inherent to this specific disease group, because it allows for exploration of findings from different angles and at different levels. Although randomised controlled trials are seen as the golden standard of research evidence, conducting this type of research was not considered feasible. One reason for this was the low prevalence of childhood ESRD and the (presumed) difficulty in getting a powered sample. We also considered the ethical challenge associated with randomising young people with ESRD to a potentially beneficial intervention [35].

A limitation of our study is the lack of an appropriate comparison group. In 2012, 518 young people with chronic conditions responded to a questionnaire about self-management that contained the same measures used in this study [47]. Unfortunately, a few respondents had ESRD, so that we could not create a comparison group.

Also, a printing error in the pre-post questionnaires in 2011 led to missing data in the self-efficacy questionnaires, thereby weakening the results of the quantitative evaluation. Furthermore, the measurements in the 2011–2012 group were timed just before and after CC, not allowing for exploration of any long-term effects. However, some long-term effects were explored by comparing this group with the retrospective sample. Although they mentioned similar effects of CC in the interviews, the quantitative results showed that the latter group, which participated longer ago, was slightly less positive about the effects. Future studies should include more measurement moments after the camp to explore the long-term effects. Finally, allowing buddies to determine the final camping programme led to different activities during the two camps and a more manifested role for buddies in CC 2012, which may have influenced our findings. However, since results from both years were compared and yielded the same findings, we expect this influence to be small.

## Conclusions

Participating in CC seems to have a positive influence on self-management of young people with ESRD aged 16–25 years. Peer-to-peer support in the form of buddy-to-attendee support is very much appreciated and support from young adults is not only beneficial for adolescent attendees, but also for the young adult buddies. It is therefore recommended to keep or start organising CC for these young people. Paediatric nephrologists are encouraged to refer patients to CC and to facilitate such initiatives. Together with nephrologists in adult care, they could take on a role in selecting buddies. Also, since young people with other chronic conditions may also benefit from CC, it is advised to explore the possibilities to organise the camp for other groups as well. When organising future camps, more attention should be given to the selection and training of buddies, and to the imminent effect of over-identification in order to counteract any negative effects. Future evaluation studies could benefit from a MMR approach, the inclusion of a control group and more measurement moments.

## Abbreviations

ESRD: End-stage renal disease; CC: Camp COOL; MMR: Mixed methods research; HRQoL: Health-related quality of life.

## Competing interests

The authors declare that they have no competing interests.

## Authors’ contributions

JNTS participated in the study’s design, carried out the literature study and data collection, performed the quantitative and qualitative analyses, and drafted the manuscript. SJ coordinated the study, participated in its design, helped with data collection and qualitative analyses, and perused the manuscript for intellectual content. AvS conceived the study, participated in its design and coordination, contributed to the interpretation of the data, and reviewed the manuscript for important intellectual content. All authors read and approved the final manuscript.

## Pre-publication history

The pre-publication history for this paper can be accessed here:

http://www.biomedcentral.com/1471-2369/14/279/prepub

## Supplementary Material

Additional file 1 The questions specifically developed for this study and considering the influence of Camp COOL on the participants are presented in Additional file 1. Click here for file

## References

[B1] Stam H Hartman EE Deurloo JA Groothoff J Grootenhuis MA Young adult patients with a history of pediatric disease: impact on course of life and transition into adulthood J Adolesc Health 2006 14 4 13 10.1016/j.jadohealth.2005.03.011 16781955

[B2] Nicholas DB Picone G Selkirk EK The lived experiences of children and adolescents with end-stage renal disease Qual Health Res 2011 14 162 173 10.1177/1049732310382789 20833832

[B3] Pomaki G Delongis A Anagnostopoulou T Heininger J Can’t live with you, can’t live without you: negative family exchanges and adaptation in end-stage renal disease patients J Health Psychol 2011 14 3 520 529 10.1177/1359105310393543 21224329

[B4] Jansen DL Heijmans M Rijken M Kaptein AA The development of and first experiences with a behavioural self-regulation intervention for end-stage renal disease patients and their partners J Health Psychol 2011 14 274 283 10.1177/1359105310372976 20733011

[B5] Kiberd JA Acott P Kiberd BA Kidney transplant survival in pediatric and young adults BMC Nephrol 2011 14 54 10.1186/1471-2369-12-54 21982270 PMC3198885

[B6] Icard PF Hower SJ Kuchenreuther AR Hooper SR Gipson DS The transition from childhood to adulthood with ESRD: educational and social challenges Clin Nephrol 2008 14 1 7 10.5414/CNP69001 18218310

[B7] Groothoff JW Grootenhuis MA Offringa M Stronks K Hutten GJ Heymans HS Social consequences in adult life of end-stage renal disease in childhood J Pediatr 2005 14 512 517 10.1016/j.jpeds.2004.10.060 15812455

[B8] Taylor RM Gibson F Franck LS A concept analysis of health-related quality of life in young people with chronic illness J Clin Nurs 2008 14 1823 1833 10.1111/j.1365-2702.2008.02379.x 18578756

[B9] Darbyshire P Oster C Henning P Children’s and young people’s experiences of chronic renal disease: a review of the literature, methodological commentary and an alternative proposal J Clin Nurs 2006 14 751 760 10.1111/j.1365-2702.2006.01510.x 16684171

[B10] Tjaden L Tong A Henning P Groothoff J Craig JC Children’s experiences of dialysis: a systematic review of qualitative studies Arch Dis Child 2012 14 395 402 10.1136/archdischild-2011-300639 22399673

[B11] Tong A Henning P Wong G McTaggart S Mackie F Carroll RP Craig JC Experiences and perspectives of adolescents and young adults with advanced CKD Am J Kidney Dis 2013 14 375 384 10.1053/j.ajkd.2012.09.024 23312724

[B12] Jansen DL Grootendorst DC Rijken M Heijmans M Kaptein AA Boeschoten EW Dekker FW PREPARE-2 Study Group Pre-dialysis patients’ perceived autonomy, self-esteem and labor participation: associations with illness perceptions and treatment perceptions. A cross-sectional study BMC Nephrol 2010 14 35 10.1186/1471-2369-11-35 21138597 PMC3019121

[B13] Grootenhuis MA Stam H Last BF Groothoff JW The impact of delayed development on the quality of life of adults with end-stage renal disease since childhood Pediatr Nephrol 2006 14 538 544 10.1007/s00467-006-0030-9 16511685

[B14] Maurice-Stam H Verhoof EJ Caron HN Grootenhuis MA Are survivors of childhood cancer with an unfavourable psychosocial developmental trajectory more likely to apply for disability benefits? Psycho-Oncol 2011 14 708 714 10.1002/pon.2112 22213575

[B15] Pendley JS Kasmen LJ Miller DL Donze J Swenson C Reeves G Peer and family support in children and adolescents with type 1 diabetes J Pediatr Psychol 2002 14 429 438 10.1093/jpepsy/27.5.429 12058007

[B16] Hughes J Wood E Smith G Exploring kidney patients’ experiences of receiving individual peer support Health Expect 2009 14 396 406 10.1111/j.1369-7625.2009.00568.x 19691464 PMC5060506

[B17] Waldrip AM Malcolm KT Jensen-Campbell LA With a little help from your friends: the importance of high-quality friendships on early adolescent adjustment Soc Dev 2008 14 832 852 10.1111/j.1467-9507.2008.00476.x

[B18] Wells F Ritchie D McPherson AC’It Is life threatening but I don’t mind’. A qualitative study using photo elicitation interviews to explore adolescents’ experiences of renal replacement therapies Child Care Health Dev 2013 14 602 612 10.1111/j.1365-2214.2012.01399.x 22676493

[B19] Olsson CA Boyce MF Toumbourou JW Sawyer SM The role of peer support in facilitating psychosocial adjustment to chronic illness in adolescence Clin Child Psychol Psychiatry 2005 14 78 87 10.1177/1359104505048793

[B20] Epstein I Stinson J Stevens B The effects of camp on health-related quality of life in children with chronic illnesses: a review of the literature J Pediatr Oncol Nurs 2005 14 89 103 10.1177/1043454204273881 15695351

[B21] Bekesi A Torok S Kokonyei G Bokretas I Szentes A Telepoczki G European KG Health-related quality of life changes of children and adolescents with chronic disease after participation in therapeutic recreation camping program Health Qual Life Outcomes 2011 14 43 10.1186/1477-7525-9-43 21672254 PMC3141371

[B22] Kiernan G Gormley M MacLachlan M Outcomes associated with participation in a therapeutic recreation camping programme for children from 15 European countries: Data from the ’Barretstown Studies’ Soc Sci Med 2004 14 903 913 10.1016/j.socscimed.2003.12.010 15186893

[B23] Shepanski MA Hurd LB Culton K Markowitz JE Mamula P Baldassano RN Health-related quality of life improves in children and adolescents with inflammatory bowel disease after attending a camp sponsored by the Crohn’s and Colitis Foundation of America Inflamm Bowel Dis 2005 14 164 170 10.1097/00054725-200502000-00010 15677910

[B24] Moons P Barrea C Suys B Ovaert C Boshoff D Eyskens B Vandenrijn C Sluysmans T Improved perceived health status persists three months after a special sports camp for children with congenital heart disease Eur J Pediatr 2006 14 767 772 10.1007/s00431-006-0171-7 16718473

[B25] Warady BA Therapeutic camping for children with end-stage renal disease Pediatr Nephrol 1994 14 387 390 10.1007/BF00866373 7917873

[B26] Cushner-Weinstein S Berl M Salpekar JA Johnson JL Pearl PL Conry JA Kolodgie M Scully A Gaillard WD Weinstein SL The benefits of a camp designed for children with epilepsy: evaluating adaptive behaviors over 3 years Epilepsy Behav 2007 14 170 178 10.1016/j.yebeh.2006.10.007 17145202

[B27] Torok S Kokonyei G Karolyi L Ittzes A Tomcsanyi T Outcome effectiveness of therapeutic recreation camping program for adolescents living with cancer and diabetes J Adolesc Health 2006 14 445 447 10.1016/j.jadohealth.2005.12.018 16919812

[B28] Hackett J Johnson B Shaw KL McDonagh JE Friends united: an evaluation of an innovative residential self-management programme in adolescent rheumatology Br J Occup Ther 2005 14 567 573

[B29] Briery BG Rabian B Psychosocial changes associated with participation in a pediatric summer camp J Pediatr Psychol 1999 14 183 190 10.1093/jpepsy/24.2.183 10361401

[B30] Sawin KJ Lannon SL Austin JK Camp experiences and attitudes toward epilepsy: a pilot study J Neurosci Nurs 2001 14 57 64 10.1097/01376517-200102000-00008 11233363

[B31] Bluebond-Langner M Perkel D Goertzel T Pediatric cancer patients’ peer relationships: the impact of an oncology camping experience J Psychosoc Oncol 1991 14 67 80

[B32] Cheung R Young Cureton V Canham DL Quality of life in adolescents with type 1 diabetes who participate in diabetes camp J Sch Nurs 2006 14 53 58 10.1177/10598405060220010901 16435931

[B33] Gillard A Witt PA Watts CE Outcomes and processes at a camp for youth with HIV/AIDS Qual Health Res 2011 14 1508 1526 10.1177/1049732311413907 21709127

[B34] O’Mahar K Holmbeck GN Jandasek B Zukerman J A camp-based intervention targeting independence among individuals with spina bifida J Pediatr Psychol 2010 14 848 856 10.1093/jpepsy/jsp125 20026569 PMC2950808

[B35] Moola FJ Faulkner GEJ White L Kirsh JA The psychological and social impact of camp for children with chronic illnesses: a systematic review update Child Care Health Dev 2013 doi:10.1111/cch.12114 10.1111/cch.12114 25250399

[B36] Walker DA Pearman D Therapeutic recreation camps: an effective intervention for children and young people with chronic illness? Arch Dis Child 2009 14 401 406 10.1136/adc.2008.145631 19139032

[B37] Hunter HL Rosnov DL Koontz D Roberts MC Camping programs for children with chronic illness as a modality for recreation, treatment, and evaluation: an example of a mission-based program evaluation of a diabetes camp J Clin Psychol Med S 2006 14 64 77 10.1007/s10880-005-9006-3

[B38] Creswell JW Research design: Qualitative, quantitative and mixed methods 2003 London: SAGE

[B39] O’Cathain A Murphy E Nicholl J Why, and how, mixed methods research is undertaken in health services research in England: a mixed methods study BMC Health Serv Res 2007 14 85 10.1186/1472-6963-7-85 17570838 PMC1906856

[B40] O’Cathain A Murphy E Nicholl J The quality of mixed methods studies in health services research J Health Serv Res Polic 2008 14 92 98 10.1258/jhsrp.2007.007074 18416914

[B41] Clark JP Godlee F, Jefferson T How to Peer Review a Qualitative Manuscript Peer Review in Health Sciences 2003 Second London: BMJ Books 219 235

[B42] Ware JE Memorandum: Users of the Medical Outcomes Study (MOS) 20-Items Short-Form Health Survey (SF-20) 1989 Santa Monica Calif: The Rand Co

[B43] Schwarzer R Jerusalem M Weinman J, Wright S, Johnston M Generalized Self-Efficacy scale Measures in health psychology: A user’s portfolio Causal and control beliefs 1995 Windsor, UK: NFER-NELSON 35 37

[B44] Van Staa AL On Your Own Feet: Adolescents with Chronic Conditions and Their Preferences and Competencies for Care (Doctoral Dissertation) 2012 Rotterdam: Rotterdam University

[B45] Schmidt S Peterson C Mühlan H The DISABKIDS Questionnaires-Handbook Incl. CD-Rom 2006 Lengerich: Pabst Science Publishers

[B46] Donkervoort M Wiegerink DJ van Meeteren J Stam HJ Roebroeck ME Transition Research Group South West N Transition to adulthood: validation of the Rotterdam transition profile for young adults with cerebral palsy and normal intelligence Dev Med Child Neurol 2009 14 53 62 10.1111/j.1469-8749.2008.03115.x 19021680

[B47] Sattoe JNT Hilberink SR van Staa A Bal R Lagging behind or not? Four distinctive social participation patterns among young adults with chronic conditions J Adol Health 2013 Epub ahead of print. doi: 10.1016/j.jadohealth.2013.09.017 10.1016/j.jadohealth.2013.09.017 24280304

[B48] Plante WA Lobato D Engel R Review of group interventions for pediatric chronic conditions J Pediatr Psychol 2001 14 435 453 10.1093/jpepsy/26.7.435 11553698

[B49] Woods K Mayes S Bartley E Fedele D Ryan J An evaluation of psychosocial outomes for children and adolescents attending a summer camp for youth with chronic illness Child Health Care 2013 14 86 98

[B50] Bandura A Self-Efficacy: The Exercise of Control 1997 New York: W.H. Freeman and Company

[B51] Brunier G Graydon J Rothman B Sherman C Liadsky R The psychological well-being of renal peer support volunteers J Adv Nurs 2002 14 40 49 10.1046/j.1365-2648.2002.02144.x 11895529

[B52] Rhee H McQuillan BE Belyea MJ Evaluation of a peer-Led asthma self-management program and benefits of the program for adolescent peer leaders Respir Care 2012 14 2082 2089 10.4187/respcare.01488 22710616 PMC3521097

[B53] Dennis CL Peer support within a health care context: a concept analysis Int J Nurs Stud 2003 14 321 332 10.1016/S0020-7489(02)00092-5 12605954

[B54] Embuldeniya G Veinot P Bell E Bell M Nyhof-Young J Sale JE Britten N The experience and impact of chronic disease peer support interventions: A qualitative synthesis Patient Educ Couns 2013 14 3 12 10.1016/j.pec.2013.02.002 23453850

[B55] Bartholomew LK Parcel GS Kok G Gottlieb NH Fernandez ME Planning Health Promotion Programs: An Intervention Mapping Approach 2011 3 San-Francisco: Jossey-Bass

