# Supplementary material for: Effective peer-to-peer support for young people with end-stage renal disease: a mixed methods evaluation of Camp COOL
Source: BMC Nephrol. 2013 Dec 21;14:279. doi: 10.1186/1471-2369-14-279 (PMC3878094; doi:10.1186/1471-2369-14-279)
Supplement: Additional file 1 — The questions specifically developed for this study and considering the influence of Camp COOL on the participants are presented in Additional file 1. [file 1471-2369-14-279-S1.docx]

**Additional file 1**

*Questions considering: Perceived influence on living with the condition (of Camp COOL)*

Indicate which of the boxes below best describes your position.

|  | Completely Disgree | Disgree | Somewhat Agree | Agree | Completely Agree |
| --- | --- | --- | --- | --- | --- |
| After participating in Camp COOL, I am more capable in dealing with the physical limitations my condition and my treatment entail. | □ | □ | □ | □ | □ |
| After participating in Camp COOL, I feel more positive about living with my condition and my treatment. | □ | □ | □ | □ | □ |
| After participating in Camp COOL, I will start living a healthier life (for example: *Not drinking when going out, taking my medication on time, and paying more attention to my fluid restriction*). | □ | □ | □ | □ | □ |
| After participating in Camp COOL, I've become more knowledgeable about my condition and my treatment. | □ | □ | □ | □ | □ |
| After participating in Camp COOL, I've become more independent (for example*: I'll be able go to the doctor for consultation by myself*) | □ | □ | □ | □ | □ |
| After participating in Camp COOL, I've gained more confidence (for example: *I'll be more proactive in asking my doctor questions*) | □ | □ | □ | □ | □ |
| After participating in Camp COOL, I'll be better at connecting with people socially. | □ | □ | □ | □ | □ |
| After participating in Camp COOL, I'm more informed about what it means to make the transfer to a hospital or department for adults. | □ | □ | □ | □ | □ |
| After participating in Camp COOL, I'll be better suited to making the transfer to a hospital or department for adults (*if you have already made this transfer, leave the boxes blank*). | □ | □ | □ | □ | □ |
| After participating in Camp COOL, I feel I'm more courageous (for example: *I'll be able to tell my boss I want to work more / less more easily*) | □ | □ | □ | □ | □ |

*Questions considering: value of the buddy-attendant concept*

*For attendants:*

Indicate which of the boxes below best describes your position.

|  | Completely Disgree | Disagree | Somewhat Agree | Agree | Completely Agree |
| --- | --- | --- | --- | --- | --- |
| At Camp COOL 2012, I learnt the most from the buddies. | □ | □ | □ | □ | □ |
| I liked the fact that there were buddies present at Camp COOL, who also suffer from a kidney condition. | □ | □ | □ | □ | □ |

*For buddies:*

Indicate which of the boxes below best describes your position.

|  | Completely Disgree | Disgree | Somewhat Agree | Agree | Completely Agree |
| --- | --- | --- | --- | --- | --- |
| At Camp COOL, I learnt more as a buddy than as a participant (*if you've never been a participant at Camp COOL, leave the boxes blank*) | □ | □ | □ | □ | □ |
| I would definitely recommend being a buddy at Camp COOL to other kidney patients. | □ | □ | □ | □ | □ |
